# Gender Differences in the Association of Smoking and Drinking with the Development of Cognitive Impairment

**DOI:** 10.1371/journal.pone.0075095

**Published:** 2013-10-04

**Authors:** Boyoung Park, Jonghan Park, Jae Kwan Jun, Kui Son Choi, Mina Suh

**Affiliations:** 1 National Cancer Control Institute, National Cancer Center, Goyang-si, Korea; 2 Department of Psychiatry, Catholic University of Daegu, Daegu, Korea; Cardiff University, United Kingdom

## Abstract

Modifiable lifestyle-related factors such as smoking and alcohol drinking are associated with cognitive impairment in the elderly population but the relationships have shown various results. To evaluate the relationship of alcohol drinking and smoking in the early 60 s with the risk of developing incident cognitive impairment. In 1999, we evaluated cognitive function, smoking, and drinking status in 3,174 inhabitants aged 60–64 years in a rural area of Korea, with a follow-up assessment of cognitive function 7 years later. A total of 1,810 individuals who did not show cognitive impairment at baseline were included. A stratified analysis was applied to evaluate how smoking and alcohol drinking affected the risk of developing cognitive impairment based on gender. Current smokers showed a higher risk for developing cognitive impairment than did never smokers (odds ratio [OR], 1.53; 95% confidence interval [CI], 1.09–2.15). The OR for female current smokers compared with never smokers was 1.62 (95% CI, 1.05–2.52), and smokers with higher pack-years were more likely to develop cognitive impairment than never smokers, showing a dose–response relationship (*P* for trend = 0.004). Frequent alcohol consumption increased the risk of developing cognitive impairment (OR, 1.68; 95% CI, 1.01–2.78), and a dose–response relationship was observed among male subjects (*P* for trend = 0.044). Infrequent drinking in females decreased the odds of developing cognitive impairment (OR, 0.67; 95% CI, 0.42–1.00), whereas frequent drinking tended to increase the odds, although this trend was not significant, suggesting a U-shaped relationship. Although the sample was small for some analyses, especially in female, our data suggest that smoking and drinking in the early 60 s are associated with a risk of developing cognitive impairment, and this relationship is characterized by gender differences.

## Introduction

The aging population in Korea has been growing rapidly. The population aged ≥65 years, who are defined as “elderly”, constituted 3.8% of the population in 1980 and 11.0% in 2010; this group is expected to be 38.2% of the total Korean population by 2050. A social burden related to geriatric psychiatry problems has been emerging as an important concern in this rapidly aging society. Cognitive impairment is a major mental health problem in the aged population; this includes dementia, from which 10% of the elderly population in Korea suffers [1]. Therefore, it is important to establish strategies to prevent or slow down the progression of cognitive impairment in the elderly population by identifying modifiable risk factors.

Many studies have suggested that modifiable lifestyle-related factors such as smoking and alcohol drinking are associated with cognitive impairment or dementia in the elderly population. However, many of these studies adopted cross-sectional designs or focused on the elderly population aged ≥65 years and had relatively short-term follow-up [2], [3]. Although the progression of aging is gradual, and no distinct division is observed between those aged ≥65 years and those <65 years, only a few studies have focused on earlier lifestyle factors and included long-term follow-up as the processes of dementia/cognitive impairment develop [4]. Considering that subjects in their early 60 s experience many social, physical, and emotional changes, such as retirement and related changes, changes in mental health and its associated factors need to be followed during this time.

When health effects of smoking or drinking were studied, gender differences should be considered. Gender differences in metabolism may lead to gender-specific consequences of drinking/smoking frequency, quantity consumed, average amount consumed, and drinking/smoking pattern [5], [6]. Although several review studies have investigated the effects of smoking and drinking on cognitive impairment, they did not focus the gender differences although there have been inconsistencies in previous articles [2], [7]–[9]. To investigate causal effects between smoking, drinking and cognitive impairment, prospective study which could avoid selection bias and recall bias is more appropriate than cross-sectional or case-controls studies [10]. However, as the authors’ knowledge, no longitudinal or prospective studies have considered the relationship of alcohol consumption and smoking with cognitive impairment in a Korean population.

Therefore, in this prospective community-based study, we evaluated the relationship of alcohol drinking and smoking in the early 60 s with the risk of developing cognitive impairment during a 7-year follow-up, after adjusting for associated covariates such as in Korea.

## Materials and Methods

### Study Population

The study population was all inhabitants aged 60–64 years living in Dalseong County, a rural area located in South Korea, in 1999. This area covers 426.92 km^2^, and 144,487 residents lived in 47,074 households at the end of 1999. Of the 3,827 inhabitants 60–64 years old who were identified by matching the national resident registration list to actual birth dates and residence in the community, 34 were institutionalized, and contact was attempted with 3,793. Among these, 191 were absent for all three visits, 402 refused to participate, and 26 did not finish; thus 3,174 subjects completed the first evaluation, for an overall participation rate of 82.9%.

Follow-up evaluations were conducted in 2006. Of the 3,174 who participated in the first evaluation, 945 were lost to follow-up because they were unreachable (*n* = 93), declined (*n* = 93), or did not finish (*n* = 26); 474 moved out of the community, and 259 died before they were contacted a second time. Finally, 2,229 subjects participated in the follow-up screening, for a follow-up rate of 70.2%.

We selected subjects without cognitive impairment at baseline by excluding 382 subjects who showed cognitive impairment at the first evaluation and 37 who did not have the results of their first cognitive evaluation. Therefore, 1,810 participants were included in the analysis for this study.

### Ethics Statement

This study was approved by the institutional review board of the Catholic University of Deagu Medical Center. All participants provided verbal informed consent because nearly 33% were never educated and 22% were illiterate, therefore to obtain written consent form from all participants was impossible. Also we considered that it took nearly half hour to finish measurements including cognitive function, depression screening, functional status, socio-demographic status, and lifestyle factors and those who completed agreed to participate with their own will. We confirm that verbal consent was collected by the trained medical doctors and medical students who conducted the interviews. The institutional review board of the Catholic University of Deagu Medical Center approved this consent procedure.

### Data Collection

Face-to-face interviews were conducted by trained medical doctors and medical students. Full information about the survey was given to the subjects, and those who agreed to participate were screened for cognitive impairment, depressive symptoms, and activities of daily living. Additionally, their socio-demographic status and lifestyle factors such as gender, marital status, number of cohabitants, employment status, education, smoking, alcohol drinking, and hospitalization during the past year were recorded in 1999.

In the 1999 study, the questionnaire included current smoking status (non-smoker, ex-smoker, and current smoker). Further questions about smoking duration and the number of cigarettes smoked per day were asked of ex-smokers and current smokers. We calculated the number of pack-years for smokers using the following equation and classified the number of pack-years into two categories: low (<50%) and high (≥50%).
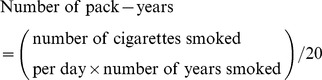



The 1999 questionnaire asked for details of current alcohol drinking status and the overall frequency of alcohol consumption. Participants were classified as non-drinkers, ex-drinkers, and current drinkers. Additionally, we classified the subjects as those who never drank alcohol, those who drank infrequently (1–3 times/week), and those who drank frequently (≥4 times/week).

Cognitive functioning was assessed in 1999 and 2006 using the Korean version of the Mini-Mental State Examination (MMSE-K). Participants who scored ≤24 on the MMSE-K were defined as cognitively impaired [11], [12]. The short form of the Geriatric Depression Scale-15 was applied to screen for depression, and a score ≥8 was suggested as probable clinical depression in the Korean population, although a cutoff score of 6 has been applied in other populations [13], [14]. Functional status was measured in terms of instrumental activities of daily living (IADL) using the Lawton Scale, and IADL impairment was defined as disability on at least one of the eight items for females and at least one of six items for males.

### Statistical Analysis

Characteristics of the 1,810 subjects without cognitive impairment at baseline are presented as numbers and proportions; the characteristics of those who did and did not develop cognitive impairment were compared using the chi-square test. The independent relationships of smoking and alcohol consumption with the development of cognitive impairment during the 7-year follow-up period were investigated by multiple logistic regression analysis, and odds ratios (ORs) with 95% confidence intervals (CIs) compared to a reference group were calculated. We used non-drinkers and non-smokers as reference groups. Stratified analyses were applied to evaluate how different levels of smoking and alcohol drinking affected the risk of developing cognitive impairment for the 7 years of follow-up separately in males and females. The *p* for trend was calculated for the number of pack-years and the number of drinking per week to investigate the dose–response relationship. All analyses were adjusted for gender (only in total participants), marital status, number of cohabitants, employment status, education, hospitalization during the past year, depressive symptoms, impairment in IADL, baseline MMSE-K score, and drinking or smoking status which showed associations with development of cognitive impairment in previous studies [15], [16]. We regarded number of cohabitants and employment status as social network which could influence better cognitive function. All statistical analyses were performed using SAS software version 9.1 (SAS Institute Inc, Cary, NC, USA).

## Results

The socio-demographic, lifestyle, and clinical characteristics of the subjects according to gender were presented in Table 1. Comparison of distribution characteristics between 812 males and 998 females were significantly different.

**Table 1:** Baseline sociodemographic characteristics of the participants at baseline according to gender.

Characteristic	Male (*n* = 812)	Female (*n* = 998)	*P*-value
	*n* (%)	*n* (%)	
Marital status			
Married	792 (97.5)	665 (66.6)	<0.001
Single/widowed/divorced	20 (2.5)	333 (33.4)	
Number of cohabitants			
0	10 (1.3)	125 (12.5)	<0.001
1	347 (42.7)	434 (43.5)	
2	208 (25.6)	170 (17.0)	
3 or more	247 (30.4)	269 (27.0)	
Employment status			
No	129 (15.9)	319 (32.0)	<0.001
Yes	683 (84.1)	679 (68.0)	
Education			
No	85 (10.5)	504 (50.5)	<0.001
≤Elementary school (1–6 years)	421 (51.8)	439 (44.0)	
>Elementary school (≥7 years)	306 (37.7)	55 (5.5)	
Smoking			
Never	131 (16.1)	865 (86.7)	<0.001
Ex-smoker	223 (27.5)	19 (1.9)	
Current smoker	458 (56.4)	114 (11.4)	
Drinking			
Never	194 (23.9)	854 (85.6)	<0.001
Ex-drinker	110 (13.5)	22 (2.2)	
Current drinker	508 (62.6)	122 (12.2)	
Hospitalization during the past year (missing in 2)			
No	225 (27.7)	168 (16.9)	<0.001
Yes	586 (72.3)	829 (83.1)	
Depressive symptom (missing in 2)			
No	695 (85.6)	725 (72.8)	<0.001
Yes	117 (14.4)	271 (27.2)	
IADL impairment (missing in 4)			
No	734 (90.4)	657 (66.1)	<0.001
Yes	78 (9.6)	337 (33.9)	
MMSE-K score			
Mean (Standard deviation)	28.5 (1.4)	27.9 (1.5)	<0.001

IADL, instrumental activities of daily living; MMSE-K, Korean version of the Mini-Mental State Examination.

The characteristics of the subjects with or without cognitive impairment are presented and compared in Table 2. Of these subjects, 600 developed cognitive impairment during the 7-year follow-up. Those who developed cognitive impairment were more likely to be female, single/widowed/divorced, unemployed, less educated, smokers, and drinkers; they were also more likely to live alone and to have depressive symptoms, IADL impairment, and lower MMSE-K score (*P*<0.05).

**Table 2:** Baseline sociodemographic characteristics of the participants at baseline according to development of cognitive impairment.

Characteristic	No cognitiveimpairment at follow up(*n* = 1210)	Cognitive impairmentat follow up (*n* = 600)	*P*-value
	*n* (%)	*n* (%)	
Gender			
Male	644 (53.2)	168 (28.0)	<0.001
Female	566 (46.8)	432 (72.0)	
Marital status			
Married	1024 (84.6)	433 (72.2)	<0.001
Single/widowed/divorced	186 (15.4)	167 (27.8)	
Number of cohabitants			
0	71 (5.9)	64 (10.7)	<0.001
1	512 (42.3)	269 (44.8)	
2	275 (22.7)	103 (17.2)	
3 or more	352 (29.1)	164 (27.3)	
Employment status			
No	282 (23.3)	166 (27.7)	0.043
Yes	928 (76.7)	434 (72.3)	
Education			
No	243 (20.1)	346 (57.7)	<0.001
≤Elementary school (1–6 years)	644 (53.2)	216 (36.0)	
>Elementary school (≥7 years)	323 (26.7)	38 (6.3)	
Smoking			
Never	374 (62.3)	622 (51.4)	<0.001
Ex-smoker	52 (8.7)	190 (15.7)	
Current smoker	174 (29.0)	398 (32.9)	
Drinking			
Never	392 (65.3)	656 (54.2)	<0.001
Ex-drinker	36 (6.0)	96 (7.9)	
Current drinker	172 (28.7)	458 (37.8)	
Hospitalization during the past year (missing in 2)			
No	270 (22.4)	123 (20.5)	0.406
Yes	938 (77.6)	477 (79.5)	
Depressive symptom (missing in 2)			
No	1018 (84.2)	402 (67.1)	<0.001
Yes	191 (15.8)	197 (32.9)	
IADL impairment (missing in 4)			
No	1035 (85.7)	356 (59.5)	<0.001
Yes	173 (14.3)	242 (40.5)	
MMSE-K score			
Mean (Standard deviation)	28.4 (1.4)	27.7 (1.6)	<0.001

IADL, instrumental activities of daily living; MMSE-K, Korean version of the Mini-Mental State Examination.

Current smokers showed a higher risk for developing cognitive impairment compared with never smokers (OR, 1.53; 95% CI, 1.09–2.15), and smokers with low pack-years showed increased odds of developing cognitive impairment compared with never smokers (OR, 1.59; 95% CI, 1.13–2.24). Although smokers with high pack-years showed increased odds of cognitive impairment, this finding was not statistically significant (OR, 1.33; 95% CI, 0.87–2.03). When we investigated the effects of smoking on the development of cognitive impairment separately by gender, only females showed significant effects of smoking. The odds of female current smokers developing cognitive impairment compared with never smokers was 1.62 (95% CI, 1.05–2.52), and smokers with high pack-years were more than twice as likely to develop cognitive impairment as were never smokers (OR, 2.37; 95% CI, 1.30–4.32), showing a dose–response relationship (*P* for trend = 0.004) (Table 3).

**Table 3:** Results of multiple logistic regression analysis showing the influence of participant smoking status in their early 60(60–64 years) on cognitive impairment after a 5-year follow-up.

	Total		Males		Females	
	OR^a^	95% CI	OR^b^	95% CI	OR^b^	95% CI
Smoking at baseline						
Never	1	(Reference)	1	(Reference)	1	(Reference)
Ex-smoker	1.30	(0.82–2.06)	1.02	(0.54–1.92)	2.09	(0.73–6.00)
Current smoker	1.53	(1.09–2.15)	1.27	(0.73–2.22)	1.62	(1.05–2.52)
Number of pack-years at baseline						
Never	1	(Reference)	1	(Reference)	1	(Reference)
Low	1.59	(1.13–2.24)	1.32	(0.76–2.30)	1.27	(0.74–2.17)
High	1.33	(0.87–2.03)	1.17	(0.63–2.18)	2.37	(1.30–4.32)
* P* for trend	0.355		0.987		0.004	

OR, odds ratio; CI, cognitive impairment.

a Adjusted for gender, marital status, number of cohabitants, employment status, education, hospitalization during the past one year, depressive symptoms, impairment in instrumental activities of daily living, Korean version of the Mini-Mental State Examination score, and drinking status at baseline.

b Adjusted for marital status, number of cohabitants, employment status, education, hospitalization during the past one year, depressive symptom, impairment in instrumental activities of daily living, Korean version of the Mini-Mental State Examination score, and drinking status at baseline.

Table 4 shows the relationship between alcohol consumption and the development of cognitive impairment. Although we did not find a relationship in all subjects, frequent alcohol consumption increased the risk for developing cognitive impairment (OR, 1.68; 95% CI, 1.01–2.78), and a dose–response relationship was observed among male subjects (*P* for trend = 0.044). However, infrequent drinking decreased the odds for developing cognitive impairment in females (OR, 0.67; 95% CI, 0.42–1.00), whereas frequent drinking tended to increase the odds, although the trend was not significant, suggesting instead a U-shaped relationship.

**Table 4:** Results of multiple logistic regression analysis showing the influence of participant alcohol consumption in their early 60(60–64 years) on cognitive impairment after a 5-year follow-up.

	Total		Male		Female	
	OR^a^	95% CI	OR^b^	95% CI	OR^b^	95% CI
Drinking at baseline						
Never	1	(Reference)	1	(Reference)	1	(Reference)
Ex-drinker	1.06	(0.64–1.76)	1.66	(0.86–3.20)	0.62	(0.24–1.62)
Current drinker	1.04	(0.77–1.41)	1.53	(0.94–2.50)	0.82	(0.53–1.26)
Number of drinking per week						
Never	1	(Reference)	1	(Reference)	1	(Reference)
Infrequent	0.88	(0.63–1.23)	1.31	(0.77–2.25)	0.67	(0.42–1.00)
Frequent	1.28	(0.88–1.86)	1.68	(1.01–2.78)	1.16	(0.52–2.57)
* P* for trend	0.159		0.044		0.613	

OR, odds ratio; CI, cognitive impairment.

a Adjusted for gender, marital status, number of cohabitants, employment status, education, hospitalization during the past one year, depressive symptom, impairment in instrumental activities of daily living, Korean version of the Mini-Mental State Examination score, and smoking status at baseline.

b Adjusted for marital status, number of cohabitants, employment status, education, hospitalization during the past year, depressive symptoms, impairment in instrumental activities of daily living, Korean version of the Mini-Mental State Examination score, and smoking status at baseline.

## Discussion

Our results indicate that smoking and drinking in the early 60 s are associated with the risk of developing cognitive impairment, although significant gender differences are observed. Compared with subjects who never smoked, women who smoked in their early 60 s had an increased risk of developing cognitive impairment. The increased risk among women smokers with higher pack-years suggested the cumulative effects of lifestyle, although no significant effects were found in males. Alcohol drinking showed a U-shaped relationship with cognitive impairment in women, whereas a dose–response relationship in which higher alcohol consumption increased the risk of developing cognitive impairment was suggested in males.

Studies of the relationship between smoking and cognitive impairment have shown various results. Some proposed that smoking reduces cognitive impairment [17] and the risk for Alzheimer’s disease [18] through an effect of nicotine that inhibits amyloidosis by preventing peptide transformation [19]. Nevertheless, recent studies have suggested that the effects of smoking on cardiovascular disease also affect the risk for vascular dementia, and cognitive decline and the increased risk for Alzheimer’s disease has been attributed to the harmful effects of smoking such as oxidative stress, inflammation, and atherosclerotic processes [20]. The controversial effects of smoking on cognitive impairment and the risk for Alzheimer’s disease differ based on study design [9]. A meta-analysis of case–control studies showed 26% a protective effect of smoking on Alzheimer’s disease, whereas cohort studies showed a two-fold increased risk for Alzheimer’s disease [9]. A recent meta-analysis restricted to prospective epidemiological studies also proposed that smokers, particularly current smokers at the baseline measurement, showed an increased risk for dementia or cognitive decline compared with never smokers, but the relationship between former smoking habits and dementia is unclear [2]. The results of our study were consistent with those of previous studies. However, the effect of smoking on the development of cognitive impairment was prominent in females, whereas we found no significant effect of smoking on cognitive impairment in males. Studies have shown that women are more susceptible to the cancer-causing effects of cigarette smoking [21], although there are controversies based on study design [22], [23]. A gender difference has been observed in the effects of genetics on the association of smoking with Parkinson’s disease [24]. In our study, women were more susceptible to the development of cognitive impairment in response to smoking than were men. This may have been due to gender differences in nicotine metabolism or hormone effects on cognitive impairment. Total plasma clearance of nicotine is lower in women than that in men [5], [25], and estrogen is a neuroprotective antioxidant that increases neuronal functioning and the neurotransmitter system, presenting a global positive effect on cognitive function [26]. The estrogen level in female participants who were 60–64 years old at baseline may have been markedly decreased due to menopause, and the oxidative stress of smoking might deteriorate cognitive function more in later years.

Low to moderate alcohol consumption has a protective effect against the development of cognitive impairment, predementia, and dementia syndromes compared with effects in non-drinkers or heavy drinkers [7], [27], although a few contradictory findings exist [8]. Alcohol drinking is related to fewer brain infarcts and shows a U-shape relationship with the prevalence of white matter lesions [8]. Low to moderate alcohol intake reduces vascular risk factors by increasing prostacyclin and high-density lipoprotein concentrations and inhibiting platelet function and the generation of thromboxane A2 [3], [8]. Heavy alcohol drinking shows harmful effects on cognitive function through alcohol-related nutritional deficiencies or the neurotoxic effects of ethanol [28]. The indirect negative effect of heavy alcohol consumption also includes abnormalities in brain morphology, regional cerebral blood flow, and brain trauma [3], [8], [28]. We did not find a relationship between alcohol consumption at baseline and the development of cognitive impairment in all participants. However, gender-specific effects of alcohol consumption on the development of cognitive impairment have been suggested. Among males, a linear dose–response relationship between alcohol consumption measured by the number of drinking per week and cognitive impairment was found, but low to moderate alcohol consumption decreased the risk for cognitive impairment in females, presenting a U-shaped association. A previous study suggested that moderate alcohol consumption has a positive effect on cognitive function in females, but no significant association in males; this difference has been attributed to different patterns of drinking by gender [29]. We measured only the number of alcohol drink per week and the average quantity of consumed alcohol could not be calculated. However, the frequency of drink showed U shaped association only in female but linear association was observed in male. It might be explained the gender different pattern in alcohol consumption, especially in Korea.

Alcohol abuse is more frequent among men than among women in Korea. The odds of alcohol abuse and dependence among males compared with females is much greater in Korea than in the US [30], and the rate of alcoholism among elderly Korean men is high [31]. The greater prevalence of alcohol abuse and dependence in the male population may be attributed to the social tolerance and even encouragement of heavy drinking among men in Korea, whereas there is less tolerance for women’s drinking [30], [31]. Based on previous results, we expect that the amount of alcohol consumed among male participants was higher and that the amount of alcohol consumed even by infrequent drinkers may be heavy enough to show a linear dose–response relationship. Infrequent drinking among female participants, who were expected to drink less alcohol, was associated with a decreased risk of cognitive impairment.

This study had several limitations. Information on smoking and alcohol consumption was self-reported, which could have led to some misclassification. Although alcohol intake was likely underestimated in the population, ranking of the intake level, as we did, was valid because of consistent under- and over-reporting, and it can be expected that the classification levels were valid [32]. We only measured the frequency of alcohol drinking per week and did not measure the amount consumed. Therefore, total alcohol intake was not reflected in the classification. However, Antilla et al. classified alcohol consumption according to the overall frequency of alcohol drinking [4]. We did not consider changes in smoking and drinking pattern at the time of follow-up although adopting more healthy lifestyles could help to maintain physical health [33]. However, by considering exposure information at the baseline measurement only, we might avoid the possibility of reverse causality. The number of frequent drinkers among female in our sample was small (only 17 who developed cognitive impairment and 16 who did not develop cognitive impairment) and it might cause the insignificant effect of frequent alcohol consumption.

Participants who were cognitively impaired might forget how much they had smoked or drank, but we excluded participants showing cognitive impairment at the baseline survey, and all subjects included in the analysis were probably cognitively intact when the amount of smoking and alcohol were asked. A nonresponse bias may have occurred, but considering the high response rate (baseline participation rate, 82.9%), the effect may have been minimal. Additionally there was the possibility of selective survival related to smoking, alcohol drinking, and cognitive impairment. Although the MMSE-K has been validated as a measure of cognitive impairment in the Korean population, it is a screening tool, so measurement bias may have occurred when cognitive function was clinically assessed. Finally, there may have been uncontrolled confounding factors, even though we adjusted for several possible and known confounding factors.

Despite these limitations, this study had several strengths. This is the first study to investigate the relationship of alcohol drinking and smoking with cognitive impairment in a Korean population using a prospective study design. Considering that studies investigating the relationships between the risk of cognitive impairment and lifestyle-related factors by gender are rare, the results of this study could contribute to establishing gender-specific preventive strategies for cognitive impairment. Additionally, we investigated changes in cognitive function among subjects in their early 60 s, who experience rapid social, physical, and emotional aging.

In conclusion, although the sample was small for some analyses, especially in female, smoking and drinking in the early 60 s were associated with a risk for developing cognitive impairment, with significant gender differences. Considering the gender differences in the association between certain lifestyle-related factors and the risk of disease [29], [34], further studies with larger sample size on gender differences and risk factors for cognitive impairment are needed to establish tailored lifestyle modification strategies to effectively prevent cognitive impairment.
